# COVID-19: A Geriatric Emergency

**DOI:** 10.3390/geriatrics5020024

**Published:** 2020-04-26

**Authors:** Virginia Boccardi, Carmelinda Ruggiero, Patrizia Mecocci

**Affiliations:** Section of Gerontology and Geriatrics, Department of Medicine, University of Perugia, Santa Maria della Misericordia Hospital, 06132 Perugia, Italy

**Keywords:** aging, older, COVID-19, frailty, geriatrics

## Abstract

The older Italian population is posing a challenge in the number of deaths for coronavirus disease 2019 (COVID-19). According to previous data from China, pre-existing health conditions dramatically increase the risk of dying from COVID-19. The presence of multiple diseases in older patients may be considered as a mark of frailty, which increases the person’s vulnerability to stress and impairs the multisystemic compensatory effort to restore homeostasis. The clinical complexity associated with the management of frailty may increase the risk of complications during infection as well as the lack of the early recognition of atypical symptoms. There is an urgent need to share expertise and clinical management skills with geriatricians as well as the need for early diagnosis to start treatment at the earliest convenience in the community, with the aim to avoid the collapse of intensive care units.

The recent outbreak of coronavirus disease 2019 (COVID-19) started in Wuhan, China, and has become a public health emergency of international concern. It has already been reported that this novel coronavirus shows a higher impact on older persons, in particular, on subjects with a higher comorbidity burden. In fact, due to the age-related changes in the immune system associated with multimorbidity, older adults are at a significantly increased risk from COVID-19 complications. After the virus has begun spreading rapidly worldwide, Italy has been the most heavily affected country after China. The average life expectancy for all Italians at birth is estimated at 82.7 years, the fourth highest in the world, and the second-highest in Europe [1]. The last data released from the Italian National Health Institute (ISS), on 12 April, showed 18,366 dead subjects positive to COVID-19, with the most (83.4%) over 70 years of age and 51.9% in the oldest old age group (over 80 years) [2]. The mortality rate is significantly higher among the elderly with pre-existing health conditions, with hypertension, cardiovascular diseases, and diabetes as the most common. This aspect may explain the high rate of mortality in Italy, a country forced to deal with chronic diseases. As compared with other European countries, the Italian population has become more long-lived with chronic diseases, which increases the individual’s vulnerability to stress and impairs the multisystemic compensatory effort to restore homeostasis [3]. Thus, the gain of more years in disability and dependence have also increased the fragility of healthcare services [4]. Another aspect that may explain the high rate of infection in the oldest group of the Italian population is the co-housing of more generations who live under the same roof, accelerating the spread of the virus from younger to older subjects, both parents and grandparents. Thus, the untested, unrecognized, infected, and asymptomatic young may represent the innocent cause for the Italian coronavirus crisis. 

This is a historical moment where many colleagues, such as anaesthesiologists, specialists in infectious disease, and internists find themselves facing a high percentage of older and frail patients affected by COVID-19. The clinical complexity and the challenges associated with the management of frailty while experiencing COVID-19 interstitial pneumonia may increase the risk of complications such as delirium, acute respiratory distress syndrome (ARDS), bacterial superinfections, sepsis, and septic shock. In this context, geriatric expertise is fundamental. Geriatricians have already faced these difficulties in managing older patients during the seasonal flu peaks, but now there is one substantial difference in the management of older adults with COVID-19 infection: the need for the early recognition of atypical symptoms and signs that are so common at this age, when their clinical expression is what we define as geriatric syndromes. The activation of already well established multi-domain interventions, provided in a timely and tailored fashion, might have a dramatic impact on a positive outcome. Geriatricians are ready to share their expertise and clinical management skills to fight the multisystemic derangements triggered by COVID-19. They have experimented and learned how important holistic and comprehensive patient management is, taking into consideration drug interactions, water balance, oxygenation, nutrition, pain control, and early mobilization. The multidimensional evaluation is critical for fighting COVID-19 infection in the old-aged, but too often, the traditional clinical approach is still prevalent. In frail old persons, where immunosenescence confuses the clinical presentation, an expression of classical pathognomonic symptoms (fever, cough, and dyspnea) cannot be expected: it would be a medical bankruptcy. 

Another dangerous situation faced by hospitals is the admission of old age people with severe diseases (myocardial infarction, heart failure, stroke, fractures) at a too-late stage because of the fear of staying in a place considered at risk for COVID-19 infection. This situation may also contribute to an increase in the mortality rate. Given the testing lags and the proportion of asymptomatic infected people, it is impossible to know the rate of infection. Still, these circumstances make infection control so difficult to contain. Therefore, there is an urgent need for early diagnosis through swabs or rapid blood tests for the population to start treatment at the earliest convenience. Although hospitals have rapidly created COVID-19 units, it has been challenging to protect old age subjects from exposure in other care settings. In addition, nursing homes nowadays are dealing with severe difficulties: relatives and visitors are banned from these facilities as part of lockdown measures, there is a shortage of medical supplies, and many healthcare professionals have been placed under quarantine because they have been found to be infected. Furthermore, social distancing measures, such as the prohibition of group activities, leading to the closing of day-care centres, and the impossibility of staying outside, have dramatically worsened the management and control of patients with disabilities or dementia, with a detrimental effect on caregiver distress and burnout. 

All these problems must be considered soon if we want to protect older people from death, health status decline, and severe disability. Taking care of the frail subjects is the most reliable sign of humanity and civilization.
